# NuSAP governs chromosome oscillation by facilitating the Kid-generated polar ejection force

**DOI:** 10.1038/ncomms10597

**Published:** 2016-02-03

**Authors:** Chenyu Li, Chenyi Xue, Qiaoyun Yang, Boon Chuan Low, Yih-Cherng Liou

**Affiliations:** 1 Department of Biological Sciences, Faculty of Science, National University of Singapore, 14 Science Drive 4, Singapore 117543, Republic of Singapore; 2 Cardiovascular Institute, Perelman School of Medicine, University of Pennsylvania, Philadelphia, Pennsylvania 19104, USA; 3 Mechanobiology Institute, National University of Singapore, Singapore 117411, Republic of Singapore; 4 Graduate School for Integrative Sciences and Engineering, National University of Singapore, Singapore 117573, Republic of Singapore

## Abstract

In vertebrate cells, chromosomes oscillate to align precisely during metaphase. NuSAP, a microtubule-associated protein, plays a critical role in stabilizing spindle microtubules. In this study, we utilize 3D time-lapse live-cell imaging to monitor the role of NuSAP in chromosome oscillation and identify NuSAP as a novel regulator of the chromokinesin, Kid. Depletion of NuSAP significantly suppresses the amplitude and velocity of chromosome oscillation. We analyse the effects of NuSAP and Kid depletion in monopolar and bipolar cells with or without kinetochore microtubule depletion. Twelve postulated conditions are deciphered to reveal the contribution of NuSAP to the polar force generated at kinetochore microtubules and to the regulation of the polar ejection force generated by Kid, thus revealing a pivotal role of NuSAP in chromosome oscillation.

## Abstract (web summary)

During metaphase, alignment of chromosomes is facilitated by oscillations driven by the chromokinesin Kid. Here Li *et al*. show that the microtubule-associated protein NuSAP is a novel regulator of Kid, regulating the amplitude and velocity of chromosome oscillation.

## Graphical abstract

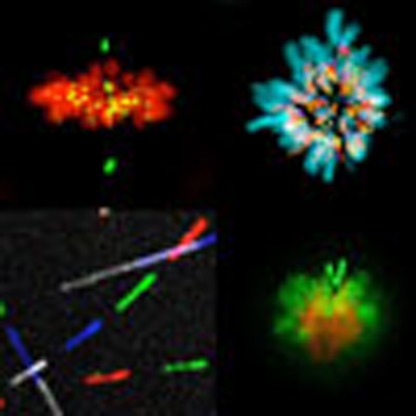

Chromosome oscillation is a common feature of metaphase in most eukaryotic cells^1^. The movement of chromosomes can be polewards or anti-polewards, referring to the direction of movement towards the pole or away from the pole^2^. Poleward motion is produced by the polar force (PF), which is predominantly generated by the depolymerization of kinetochore microtubules (kMTs)^3^, while anti-poleward motion is produced by the polar ejection force (PEF), which is dependent on motor proteins sliding along the chromosome arms at interpolar microtubules (iMTs)^4^. Although a number of studies have focused on two-dimensional kinetochore (KT) behaviour^5^,^6^,^7^,^8^ and the biophysical prediction of KT movement^9^,^10^,^11^,^12^, the underlying molecular mechanism of KT oscillation is still largely unknown.

Microtubule-associated proteins (MAPs) play vital roles in regulating chromosome oscillation by tightly maintaining both the dynamics of kMTs and the surface properties of iMTs^13^. NuSAP (Nucleolar and Spindle-Associated Protein)^14^, a RanGTP-regulated MAP, bundles microtubules^15^ and links them to chromosomes^16^. In addition, NuSAP regulates spindle assembly, chromosome segregation and cytokinesis^14^,^17^. The level of NuSAP protein expression is tightly regulated during the cell cycle by anaphase-promoting complex/cyclosome^18^,^19^ and is upregulated in several types of cancer^20^,^21^,^22^,^23^,^24^,^25^. However, the function of NuSAP in chromosome oscillation has not yet been elucidated.

Human chromokinesins are plus-end-directed motors contributing to anti-poleward movement^26^,^27^,^28^. These include Kid (kinesin-like DNA-binding protein), which contains an N-terminal microtubule-binding domain and a C-terminal chromosome-interacting domain^29^. Kid functions as a microtubule-based motor, generating the PEF, and regulating the orientation of chromosome arms and KT oscillation^7^,^8^,^30^. The RanGTP gradient, which regulates NuSAP localization, promotes the accumulation of Kid on chromosomes^31^. Although a functional relationship has been reported between NuMA and Kid in spindle morphology and chromosome alignment^32^, and the microtubule localization of Kid is known to be mediated by the spindle protein CHICA^33^, the regulatory mechanism of Kid in chromosome oscillation remains unclear.

In this study, we sought to determine the role of NuSAP in chromosome oscillation. We use three-dimensional (3D) time-lapse live-cell imaging to analyse chromosome oscillation in a dynamically heterogeneous population to determine the influence of NuSAP on the Kid-generated PEF. Our results show that NuSAP plays a pivotal role in mediating chromosome oscillation through its regulation on the Kid-generated PEF during metaphase.

## Results

### NuSAP regulates chromosome alignment and orientation

**Figure 1:**
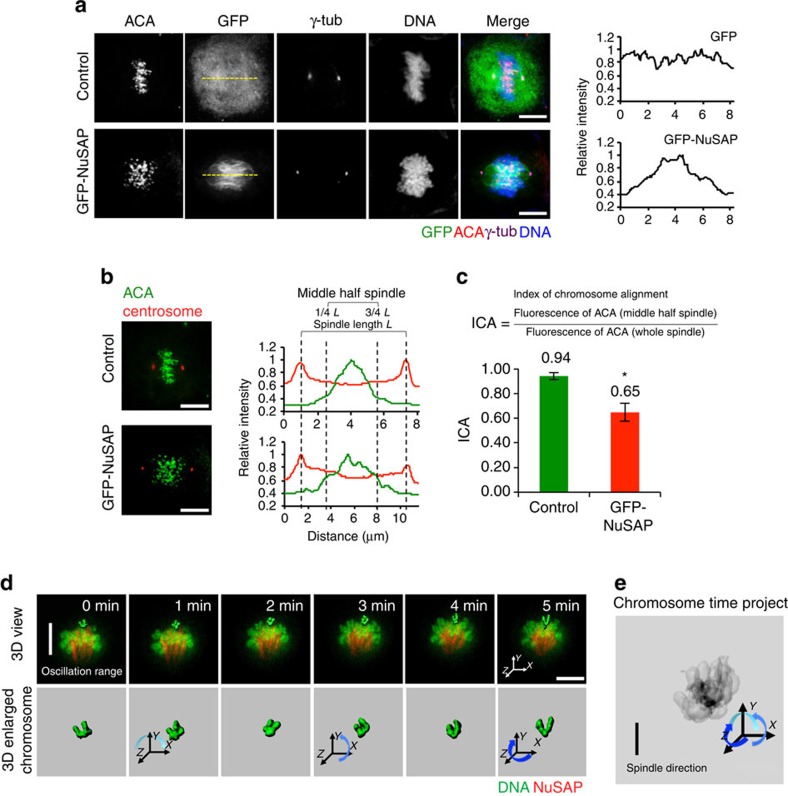
NuSAP regulates chromosome alignment and orientation during metaphase. (**a**) Fluorescent images of synchronized metaphase HeLa cells expressing GFP-NuSAP and GFP-vector (control). The line profiles of the GFP channel are presented in the graph on the right. Mitotic spindles were labelled with anti-γ-tubulin and anti-CREST, and DNA with Hoechst 333342. Scale bar, 5 μm. (**b**) Representative images using the ICA method to analyse centromere alignment in HeLa cells expressing GFP-NuSAP and GFP-vector only (control), with data represented as a line graph on the right. The distribution of ACA fluorescence within the spindle region was measured along the spindle axis following staining with anti-γ-tubulin and anti-Crest. (**c**) ICAs in HeLa cells expressing GFP-NuSAP and GFP-vector only (control) were analysed as in **b**. Data were collected from three independent experiments and error bars represent±s.d. *P<0.001, by *t*-test. (**d**) 3D time-lapse imaging and 3D single chromosome surface of chromosome oscillation in synchronized metaphase mCherry-H2B HeLa cells stably expressing GFP-NuSAP. Changes in the chromosome orientation are indicated by arrows. Scale bar, 5 μm. (**e**) Time project image representing the single chromosome misorientation.

To determine the function of NuSAP during metaphase, we first investigated the localization of GFP-NuSAP along the spindle using fluorescent imaging (Fig. 1a). Line graphs showing the intensity of signal across the spindle pole showed that NuSAP predominantly localized at the central spindle microtubules. NuSAP-overexpressing cells were also found to display a large proportion of misaligned chromosomes (Fig. 1b). To quantify the level of misalignment, we applied the index of chromosome alignment^7^, which calculates the ratio of the fluorescence of anticentromere antibody staining in the central spindle compared with the whole spindle (Fig. 1b). The index of chromosome alignment of GFP-NuSAP-overexpressing cells was significantly smaller (0.65±0.07, ±s.d. from three independent experiments) than that of the control cells (0.94±0.03), indicating a severe chromosome misalignment phenotype (Fig. 1c), which suggests that NuSAP may have a role in regulating chromosome alignment.

To determine how NuSAP disturbs chromosome congression, we used 3D time-lapse live-cell imaging to monitor the movement of chromosomes in synchronized HeLa cells stably expressing mCherry-H2B (Fig. 1d and Supplementary Movie 1). Strikingly, we found that chromosomes in cells overexpressing NuSAP displayed prominent misorientation, with the arms parallel rather than perpendicular to the spindle axis (Fig. 1d, row 1). Selected misaligned chromosomes are presented as a 3D reconstruction (row 2). The time-lapse projection for the lagging chromosome showed that the arms of the chromosome were rotating and stretching over time (Fig. 1e), suggesting that unbalanced forces on the arms were disrupting the movement of the chromosomes. Thus, upregulated NuSAP protein levels results in an imbalance of forces on the chromosome arms, resulting in chromosome misorientation.

### NuSAP interacts with Kid and regulates its localization

**Figure 2:**
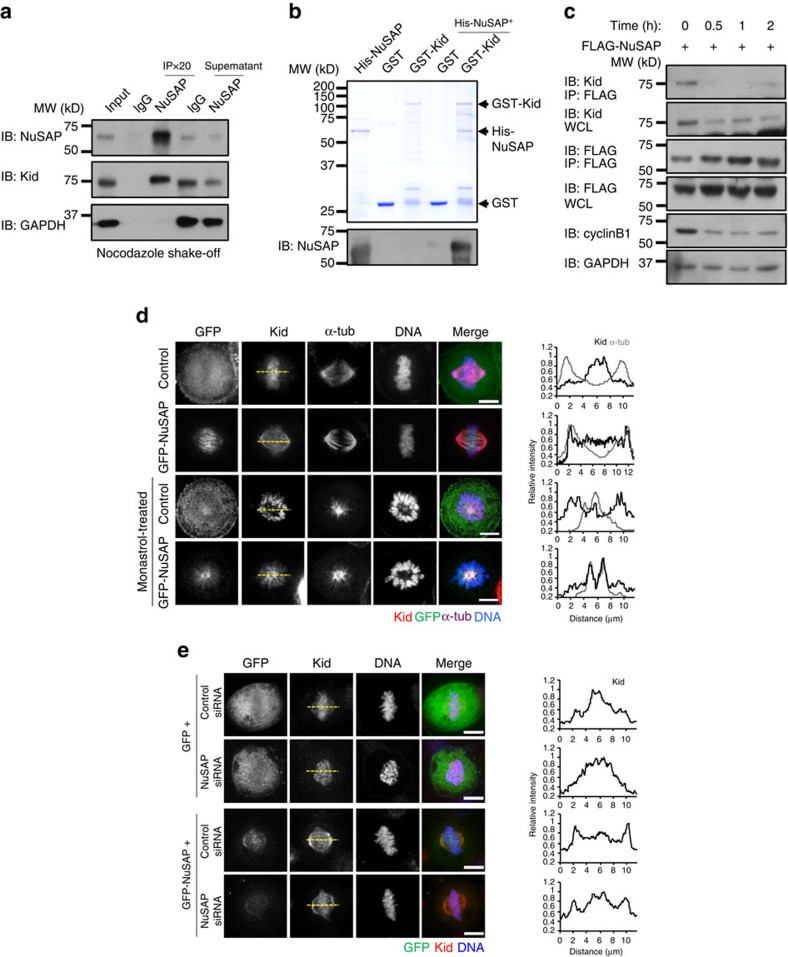
NuSAP binds Kid and regulates its localization at microtubules. (**a**) NuSAP binds Kid endogenously. Synchronized HeLa cell lysates were incubated with 2 μg rabbit IgG or NuSAP antibody bound to protein A-Sepharose beads. The immunoprecipitated proteins and supernatants were blotted with anti-Kid, anti-NuSAP and anti-GAPDH antibodies. (**b**) NuSAP binds Kid *in vitro*. Purified His-NuSAP recombinant protein was incubated with either GST or GST-Kid recombinant protein. GST was pulled down and NuSAP was blotted with an anti-NuSAP antibody. (**c**) Immunoprecipitation of NuSAP with Kid was conducted with whole-cell lysates of HeLa cells transfected with FLAG-NuSAP after release from thymidine/nocodazole arrest into fresh medium at the indicated time points. The immunoprecipitated protein and the whole-cell lysates were blotted with anti-Kid, anti-FLAG, anti-cyclin B1 and anti-GAPDH antibodies. (**d**) Kid localization at the kinetochore region in bipolar metaphase HeLa cells and monastrol-treated monopolar cells expressing GFP-NuSAP and the GFP-vector (control). Cells were stained with anti-α-tubulin and Hoechst 33342. The line profile of Kid localization (black) according to α-tubulin (grey) is represented in the right-hand graph. Scale bar, 5 μm. (**e**) Kid localization in bipolar metaphase HeLa cells transfected with control siRNA or NuSAP siRNA together with GFP-vector or GFP-NuSAP. The line profile of Kid localization (black) is represented in the right-hand graph. Scale bar, 5 μm.

The faulty orientation of chromosomes in NuSAP-overexpressing cells suggests unbalanced forces on the chromosome arms. The configuration of the chromosome pushing away from the spindle poles in *Drosophila* cells was identified as the elevation of PEF^34^. Previous studies indicate that Kid, a chromokinesin, is essential for generating the PEF at chromosome arms during chromosome oscillation^5^,^8^. This observation motivated us to investigate the interaction between NuSAP and Kid. Immunoprecipitation experiments indicated that NuSAP interacted with Kid endogenously (Fig. 2a and Supplementary Fig. 1a,b). Using purified recombinant His-tagged NuSAP and glutathione *S*-transferase (GST)-tagged Kid proteins, we confirmed this interaction by demonstrating that NuSAP could be pulled down by Kid *in vitro* (Fig. 2b). To determine the temporal nature of this interaction during the mitotic phase, cells were first arrested using thymidine/nocodazole treatments and then released into fresh medium (Fig. 2c). Immunoprecipitation at various time points post release indicated that the interaction of NuSAP with Kid occurred specifically at metaphase (Fig. 2c, lane 1).

The PEF generated by Kid during metaphase largely depends on the association of Kid with iMTs^29^,^35^. As NuSAP primarily localizes to the central spindle microtubules, we questioned whether the interaction between NuSAP and Kid affects the localization of Kid on microtubules. To address this question, we first examined the localization of endogenous Kid and NuSAP on mitotic spindles. Both Kid and NuSAP were observed to concentrate in the middle spindle (Supplementary Fig. 1c). NuSAP overexpression was found to disrupt the distribution of Kid along the spindle, with an increased fluorescent intensity along the microtubules in monopolar and metaphase cells (Fig. 2d). This observation was confirmed using live cells expressing mCherry-Kid (Supplementary Fig. 1d). Overexpression of NuSAP increased the association of Kid with microtubules, while depletion of NuSAP caused Kid to relocalize to chromosomes. To rule out the possibility of a dominant-negative effect, GFP-vector or GFP-NuSAP was expressed in either control short interfering RNA (siRNA)- or NuSAP siRNA-depleted cells (Fig. 2e). The microtubule localization of Kid could be rescued by overexpression of NuSAP (Fig. 2e, row 4). Taken together, our results indicate that NuSAP interacts with Kid at metaphase and enhances the association between Kid and microtubules.

### The depletion of NuSAP or Kid attenuates centromere movement

**Figure 3:**
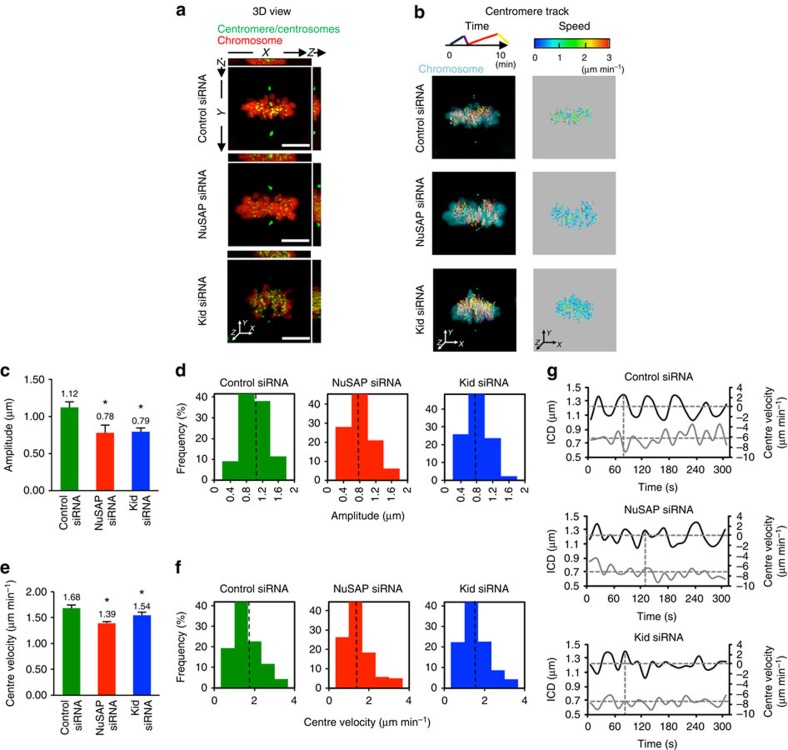
The depletion of NuSAP or Kid attenuates the amplitude and velocity of centromere movements. (**a**) Representative images of the 3D view using *xz* and *yz* projections in the control, NuSAP- or Kid-depleted synchronized metaphase mCherry-H2B HeLa cells. Centromeres were marked with GFP-CENPA and centrosomes with GFP-centrin. Scale bar, 5 μm. (**b**) Representative images of 3D centromere tracks, colour-coded for time and velocity. (**c**,**d**) Bar chart and histogram representing the average (**c**) and the distribution (**d**) of the amplitude of centromere oscillation in the control, NuSAP- or Kid-depleted metaphase cells. Error bars represent +s.e.m. *P<0.001, by *t*-test. (**e**,**f**) Bar chart and the histogram representing the average (**e**) and the distribution (**f**) of the centre velocity. Error bars represent +s.e.m. *P<0.001, by *t*-test. (**g**) Representative velocity (solid black line) and ICD (solid grey line) versus time plots of the projected centre in a sister centromere pair in control, NuSAP- and Kid-depleted metaphase cells. The sign of the velocity indicates the direction of the centromere movement. The average of the ICD (horizontal) and the minimum (horizontal) and maximum velocities (vertical) are indicated by dashed lines.

To understand how NuSAP regulates Kid function in chromosome oscillation, 3D time-lapse live-cell imaging was conducted with GFP-CENPA-labelled centromeres in synchronized HeLa cells stably expressing mCherry-H2B. Cells were transfected with control, NuSAP (Supplementary Fig. 2a–c) or Kid siRNA (Supplementary Fig. 3a) for 48 h and the metaphase cells were imaged. Representative 3D images using *xz* and *yz* projections are shown in Fig. 3a and Supplementary Movie 2. To gain insight into the KT dynamics, the centromere positions were detected and tracked in 3D over time during oscillation, maximizing the signal-to-noise ratio and minimizing phototoxicity^36^. The tracks were colour-coded over time (Fig. 3b, lane 1). Consistent with previous work^7^,^8^, the centromeres in Kid-depleted cells exhibited a slower velocity, with most tracks shown in blue and green (0-2 μm min^−1^) and fewer in red (2-3 μm min^−1^), when colour-coded for velocity, compared with the control cells (Fig. 3b, lane 2). The majority of centromere velocities in NuSAP-depleted cells were also in the 0-2 μm min^−1^ range, a similar pattern to that of Kid-depleted cells (Fig. 3b, lane 2), indicating that depletion of either NuSAP or Kid results in a decreased velocity of chromosome oscillation in metaphase cells.

To further investigate the function of NuSAP and Kid in chromosome movement, we characterized the intercentromere distance (ICD) of sister centromeres and the half-period of the 3D time-lapse tracks (Table 1). The ICDs in the NuSAP-depleted (0.69±0.09 μm) and Kid-depleted (0.71±0.10 μm) cells were not significantly changed compared with that of the control cells (0.75±0.09 μm). The average half-periods also displayed similar ranges with 32.50±3.34 s for the control siRNA cells, 32.56±3.55 s for the NuSAP-depleted cells and 31.73±4.14 s for the Kid-depleted cells (Table 1), suggesting that the stiffness between the sister centromeres was not greatly affected by the depletion of NuSAP or Kid. To investigate the effects of NuSAP and Kid depletion on the kinetic features of the oscillatory activity, we set the projected 3D displacement of the centre in a sister centromere pair as the centre point tracks over a time course to show the oscillation of a particular chromosome^11^,^36^. As shown in Fig. 3c, and consistent with previous reports^7^,^8^,^32^, the oscillation amplitude of the centre in Kid-depleted cells was dramatically decreased (0.79±0.05 μm) compared with the control cells (1.12±0.08 μm). Similarly, the oscillation amplitude in NuSAP-depleted cells was reduced to 0.78±0.11 μm (Fig. 3c). In addition, the distribution of the amplitude in the NuSAP or Kid-depleted cells showed an increase in the lower range (0-1 μm) and a decrease in the larger range (1-2 μm) compared with the control cells (Fig. 3d). As the amplitude of the oscillation is directly correlated with the PEF^37^, the significant decrease in the amplitude in the NuSAP- and Kid-depleted cells suggests a general reduction of the PEF. To analyse the dynamics of the chromosome during oscillation further, the velocity of the projected centre point in control, NuSAP- or Kid-depleted cells was also calculated. As shown in Fig. 3e, the depletion of either NuSAP (1.39±0.03 μm min^−1^) or Kid (1.54±0.06 μm min^−1^) significantly reduced the centre oscillation velocity compared with the control-depleted cells (1.68±0.06 μm min^−1^). The centromeres in the NuSAP- or Kid-depleted cells had a lower proportion in the large velocity range (2-4 μm min^−1^) than that in the control cells (Fig. 3f), indicating that the depletion of NuSAP or Kid markedly reduced the high velocity of oscillation. As the velocity varies with the position of the centromere during oscillation and the maximum velocity is reached near the equator with the minimum ICD^7^,^38^, we further investigated whether NuSAP and Kid can regulate the coupling of the velocity and the ICD versus time and position. As shown in Fig. 3g, in control cells, the maximum velocity was reached near the minimum ICD with approximately twice the frequency, consistent with a previous study^38^. However, in NuSAP- or Kid-depleted cells, the change in velocity was considerably irregular and coupling between velocity and ICD was abolished (Fig. 3g, rows 2 and 3). The function of NuSAP on chromosome oscillation was further confirmed using NuSAP siRNA (Supplementary Fig. 3a–c), and to rule out a possible dominant-negative effect, rescue experiments using mCherry-NuSAP expression in NuSAP siRNA-depleted cells were also conducted (Supplementary Fig. 3b,c). Overexpression of mCherry-NuSAP, but not mCherry vector, facilitated KT oscillation in NuSAP-depleted cells. Taken together, the data suggest that depletion of NuSAP or Kid attenuates the overall motility and coupling of centromere oscillation.

### NuSAP and Kid regulate centromere movement at iMTs

**Figure 4:**
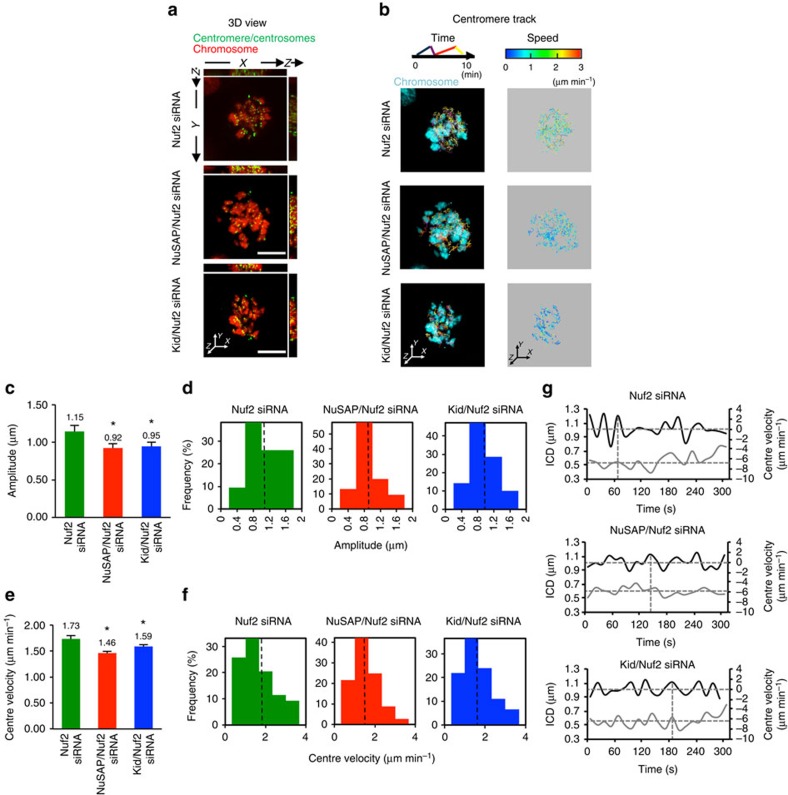
NuSAP and Kid regulate centromere movement at interpolar microtubules. (**a**) Representative images of the 3D view using *xz* and *yz* projections in Nuf2-, NuSAP/Nuf2- or Kid/Nuf2-depleted synchronized metaphase HeLa cells stably expressing mCherry-H2B. Centromeres were marked with GFP-CENPA and centrosomes with GFP-centrin. Scale bar, 5 μm. (**b**) Representative images of the 3D centromere tracks colour-coded for time and velocity. (**c**,**d**) Bar chart and histogram representing the average (**c**) and the distribution (**d**) of the amplitude of centromere oscillation in Nuf2-, NuSAP/Nuf2- or Kid/Nuf2-depleted metaphase cells. Error bars represent +s.e.m. *P<0.001, by *t*-test. (**e**,**f**) Bar chart and histogram representing the average (**e**) and the distribution (**f**) of the centre velocity. Error bars represent +s.e.m. *P<0.001 by *t*-test. (**g**) Representative velocity (black line) and ICD (grey line) versus time plots of the projected centre in a sister centromere pair in Nuf2-, NuSAP/Nuf2- or Kid/Nuf2-depleted metaphase cells. The sign of the velocity indicates the direction of centromere movement. The average of the ICD (horizontal) and the minimum (horizontal) and maximum velocities (vertical) are indicated by the dashed lines.

Our findings indicate that NuSAP and Kid tune the amplitude and velocity of centromere oscillation. However, we could not rule out the possibility that the regulation of chromosome movement by NuSAP may also result from its influence on kMTs. To examine this possibility, we depleted Nuf2 using siRNA to specifically diminish kMTs in the cells^39^, and subsequently characterized centromere oscillation in NuSAP/Nuf2 or Kid/Nuf2 double-depleted cells using Nuf2-depleted cells as a control (Supplementary Fig. 4b). The depletion of Nuf2 was further examined by immunofluorescence, which showed that KT-microtubule attachment was damaged in Nuf2-depleted cells (Supplementary Fig. 4c). The depletion of Nuf2 resulted in ∼84% of cells with misaligned chromosomes (Supplementary Fig. 4d), which is consistent with previous studies^36^,^40^,^41^. Our result indicates that Nuf2 was strongly depleted, removing most KT fibres; however, since it was not completely depleted, the residual kMTs might still be present. Representative 3D time-lapse images are shown in Fig. 4a and Supplementary Movie 3, and the centromere tracks, colour-coded for time, are shown in Fig. 4b, lane 1. The movement of centromeres in Nuf2-depleted cells displayed a faster velocity with more tracks shown in yellow-red (2-3 μm min^−1^) when colour-coded for velocity (Fig. 4b, lane 2, top). On the other hand, the velocities of centromeres in the NuSAP/Nuf2 and Kid/Nuf2 double-depleted cells were mostly in the 0-2 μm min^−1^ range (shown as blue and green in Fig. 4b, lane 2, middle and bottom), indicating that the velocity of centromere movement was dramatically decreased by co-depletion of NuSAP or Kid in Nuf2-depleted cells.

The amplitude of the centre oscillation in NuSAP/Nuf2- or Kid/Nuf2-depleted cells was dramatically reduced compared with that in Nuf2-depleted cells (Fig. 4c). In addition, the frequency of oscillations with high amplitude (1-2 μm) was significantly decreased by co-depletion with NuSAP or Kid in the Nuf2-depleted cells (Fig. 4d). The velocity of the centre was also significantly decreased in the NuSAP/Nuf2- and Kid/Nuf2-depleted cells (Fig. 4e) and showed a largely decreased proportion in the high velocity range, compared with that in Nuf2-depleted cells (2-4 μm min^−1^, Fig. 4f). The coupling of the maximum velocity with the minimum ICD was also abolished in Nuf2-, NuSAP/Nuf2- and Kid/Nuf2-depleted cells (Fig. 4g, vertical dotted lines), indicating that this linkage requires kMT attachment. The overall attenuation of oscillation in the absence of kMTs following depletion of Nuf2 suggests that the elimination of chromosome oscillation by NuSAP or Kid is predominantly on iMTs.

### NuSAP and Kid enhance KT oscillation in monopolar cells

**Figure 5:**
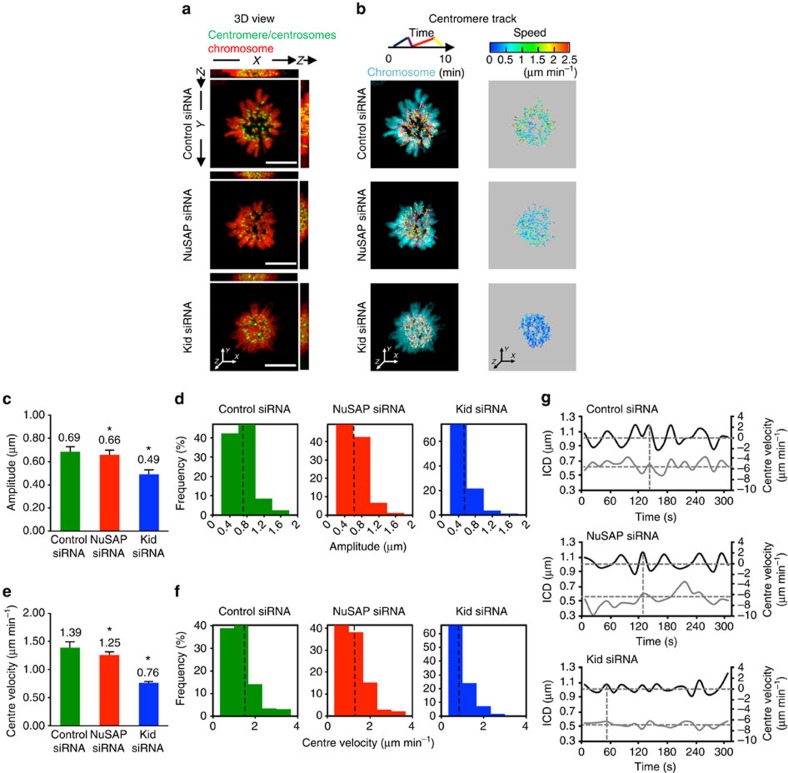
The effects of NuSAP and Kid depletion on the amplitude and velocity of centromere oscillation in monopolar cells are correlated. (**a**) Representative images of the 3D view using *xz* and *yz* projections in the control, NuSAP- or Kid-depleted monastrol-treated monopolar HeLa cells stably expressing mCherry-H2B. Centromeres were marked with GFP-CENPA and centrosomes with GFP-centrin. Scale bar, 5 μm. (**b**) Representative images of the 3D centromere tracks colour-coded for time and velocity. (**c**,**d**) Bar chart and histogram representing the average (**c**) and the distribution (**d**) of the amplitude of centromere oscillation in the control, NuSAP- or Kid-depleted monopolar cells. Error bars represent +s.e.m. *P<0.001, by *t*-test. (**e**,**f**) Bar chart and histogram representing the average (**e**) and the distribution (**f**) of the centre velocity. Error bars represent +s.e.m. *P<0.001, by *t*-test. (**g**) Representative velocity (black line) and ICD (grey line) versus time plots of the projected centre in a sister centromere pair in the control, NuSAP- or Kid-depleted monopolar cells. The sign of the velocity indicates the direction of centromere movement. The average of the ICD (horizontal) and the minimum (horizontal) and maximum velocities (vertical) are indicated by the dashed lines.

In bipolar cells, centromere oscillation is regulated by the motors, the interpolar and kMTs from two poles with opposite directions. To simplify the factors involved in chromosome oscillation further, we studied the movement of centromeres in monastrol-treated monopolar cells with both iMTs and kMTs from one spindle pole with a single direction^2^,^4^,^10^,^38^. Representative 3D time-lapse images (Fig. 5a) and colour-coded centromere tracks (Fig. 5b, lane 1) of the control, NuSAP- or Kid-depleted cells are shown in Fig. 5 (Supplementary Movie 4). The centromere tracks indicate that centromere movement in NuSAP-depleted or Kid-depleted cells was slower, with more tracks shown in blue (0-1 μm min^−1^) compared with the control cells (Fig. 5b, lane 2).

Depletion of NuSAP or Kid in monopolar cells resulted in an even more severe decrease in oscillation amplitude (Fig. 5c), with a greater proportion in the 0-1 μm range compared with the control monopolar cells (Fig. 5d). Furthermore, the velocity of the centre was significantly decreased in NuSAP- or Kid-depleted monopolar cells, with a dramatically decreased proportion in the range of 2-4 μm min^−1^ (Fig. 5e,f). We noted that the effect of Kid depletion on oscillation amplitude and velocity in monopolar cells was greater than that of NuSAP (Fig. 5e,f, lane 3). Because the PEF generated by Kid is directly related to the density of the iMTs at the function positions^12^,^37^, the higher density of microtubules in the monopolar aster structure compared with the bipolar spindle might result in an increase in the Kid-generated PEF. This is consistent with a previous finding that mono-oriented chromosome arms are immediately ejected outwards once cut free^4^. Moreover, the depletion of NuSAP may affect both the PF at kMTs and the PEF at iMTs with opposing directions, leading to a reduced overall effect on chromosome movement. The coupling of velocity and ICD was abolished in the control, NuSAP- and Kid-depleted monopolar cells (Fig. 5g), indicating that non-random centromere oscillation also requires a bipolar spindle structure. Taken together, these data suggest that both NuSAP and Kid positively affect the amplitude and velocity of centromere oscillation in monopolar cells.

### NuSAP tunes the PEF with Kid at iMTs in monopolar cells

**Figure 6:**
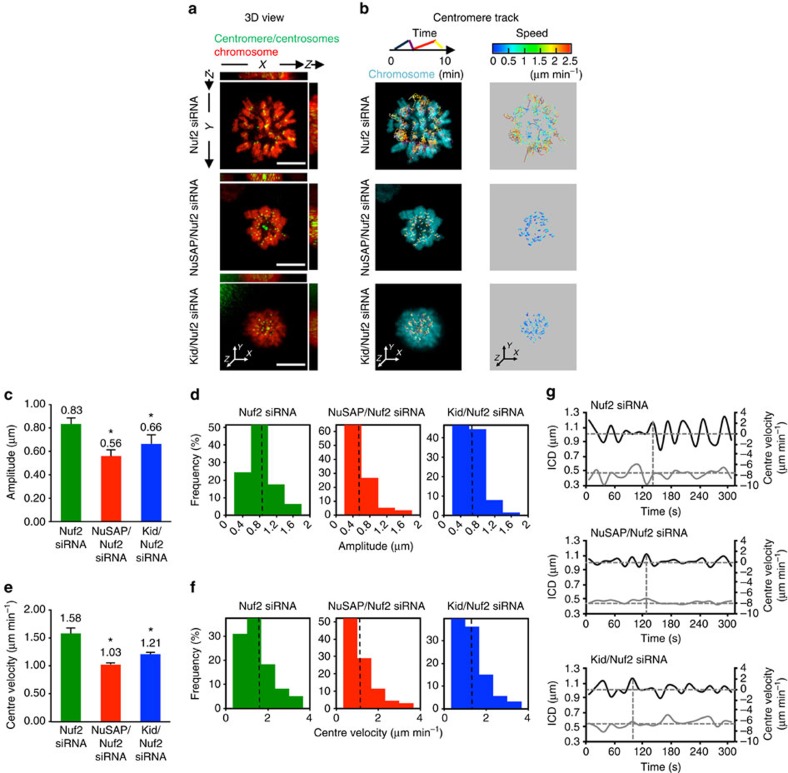
NuSAP tunes the polar ejection force with Kid at interpolar microtubules in monopolar cells. (**a**) Representative images of the 3D view using *xz* and *yz* projections in Nuf2-, NuSAP/Nuf2- or Kid/Nuf2-depleted monastrol-treated monopolar HeLa cells stably expressing mCherry-H2B. Centromeres were marked with GFP-CENPA and centrosomes with GFP-centrin. Scale bar, 5 μm. (**b**) Representative images of 3D centromere tracks colour-coded for time and velocity. (**c**,**d**) Bar chart and histogram representing the average (**c**) and the distribution (**d**) of the amplitude of centromere oscillation in Nuf2-, NuSAP/Nuf2- or Kid/Nuf2-depleted monopolar cells. Error bars represent +s.e.m. *P<0.001, by *t*-test. (**e**,**f**) Bar chart and histogram representing the average (**e**) and the distribution (**f**) of the centre velocity. Error bars represent +s.e.m. *P<0.001, by *t*-test. (**g**) Representative velocity (black line) and ICD (grey line) versus time plots of the projected centre in a sister centromere pair in the control, NuSAP- or Kid-depleted monopolar cells. The sign of the velocity indicates the direction of centromere movement. The average of the ICD (horizontal) and the minimum (horizontal) and maximum velocities (vertical) are indicated by the dashed lines.

Using experiments combining both the depletion of Nuf2 to disrupt kMTs, and monastrol treatment to form monopolar spindles, we specifically investigated the PEF on iMTs with a single direction in the Nuf2-, NuSAP/Nuf2- or Kid/Nuf2-depleted monopolar cells. The representative 3D time-lapse live-cell images and centromere tracks (Fig. 6a,b and Supplementary Movie 5) show that oscillation was dramatically retarded with a significant decrease in velocity in the NuSAP/Nuf2- and Kid/Nuf2-depleted cells (Fig. 6b, lane 2, middle and bottom).

The amplitude of oscillation at the centre point in NuSAP/Nuf2 (0.56 μm)- and Kid/Nuf2 (0.66 μm)-depleted monopolar cells was also significantly reduced compared with that in control cells (0.83 μm), as shown by the substantial decrease in both the average amplitude (Fig. 6c) and the proportion in the 1-2 μm range (Fig. 6d). Moreover, the centre velocity was significantly attenuated in NuSAP/Nuf2 (1.03 μm min^−1^)- and Kid/Nuf2 (1.21 μm min^−1^)-depleted monopolar cells compared with that in control cells (1.58 μm min^−1^; Fig. 6e,f). The significant decrease in oscillation velocity and amplitude in both the NuSAP/Nuf2- and Kid/Nuf2-depleted cells suggests that NuSAP strongly regulates the forces on iMTs, particularly the PEF generated by Kid. Compared with the depletion of Kid/Nuf2, co-depletion of NuSAP and Nuf2 had a greater effect on chromosome oscillation (Fig. 6e,f, lane 2). This indicates that NuSAP might also facilitate other PEFs at iMTs besides that generated by Kid. The linkage between velocity and ICD during oscillation was also dramatically reduced in Nuf2-, NuSAP/Nuf2- and Kid/Nuf2-depleted monopolar cells, with extremely random movements observed (Fig. 6g; vertical dotted lines). This is consistent with our previous results (Figs 3, 4, 5g) showing that kMT attachment and a bipolar spindle structure are both required for proper chromosome oscillation. Taken together, these data suggest that NuSAP governs chromosome oscillation by strongly tuning the Kid-generated PEF on iMTs.

### NuSAP governs the PEF generated by Kid during KT oscillation

**Figure 7:**
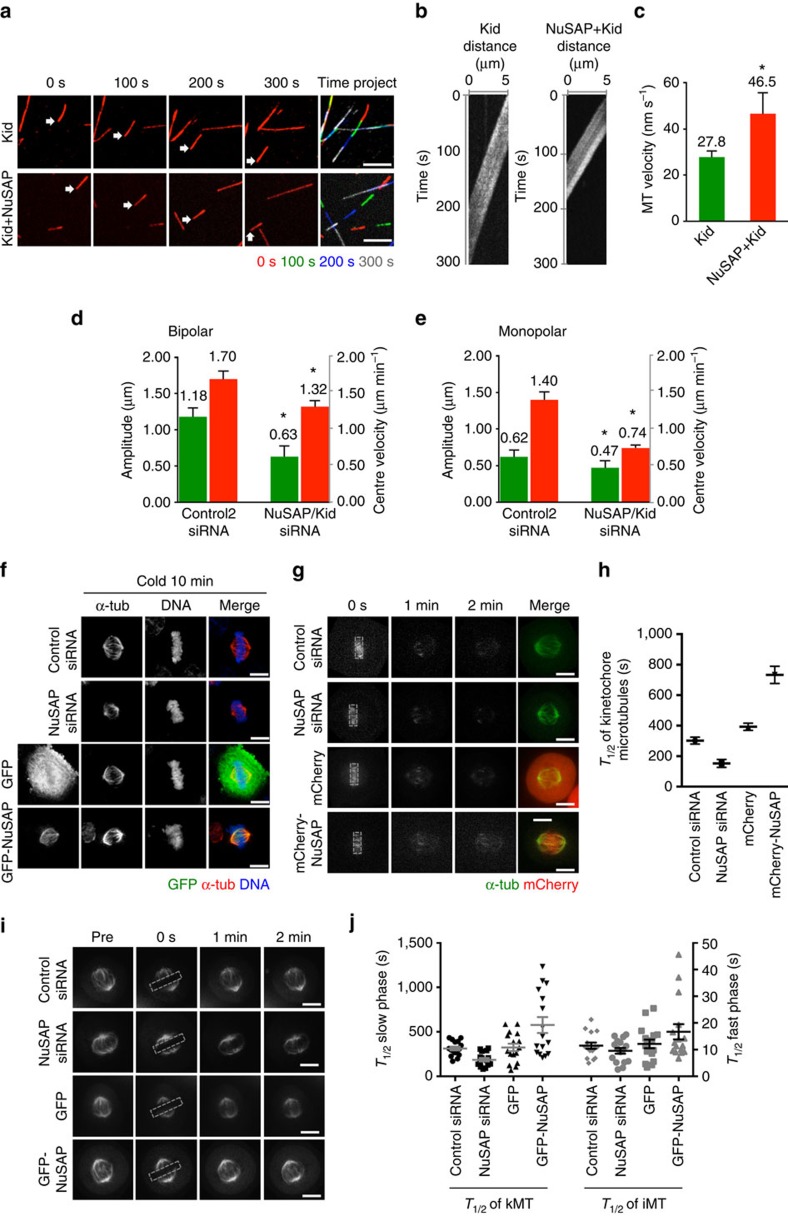
NuSAP governs Kid-generated PEF during chromosome oscillation. (**a**) Representative images and colour project of MT gliding assay with Kid or NuSAP and Kid proteins. Arrows represent one MT gliding at different time points. Images were acquired in a 2 s interval for 5 min. Scale bar, 3 μm. (**b**) Kymographs showing a sample microtubule gliding on Kid-coated surfaces in the presence or absence of NuSAP protein. (**c**) Bar chart representing the average velocity of MT gliding on Kid-coated surfaces in the presence or absence of NuSAP protein. Error bars represent±s.d. *P<0.01, by *t*-test. (**d**) Bar chart representing the average of the amplitude (black) and centre velocity (grey) of centromere oscillation in control- or NuSAP/Kid-double-depleted metaphase cells. Error bars represent +s.e.m. *P<0.001, by *t*-test. (**e**) Bar chart representing the average of the amplitude (black) and centre velocity (grey) of centromere oscillation in control- or NuSAP/Kid-double-depleted monopolar cells. Error bars represent +s.e.m. *P<0.001, by *t*-test. (**f**) The kinetochore MTs in metaphase HeLa cells transfected with control siRNA, NuSAP siRNA, GFP-vector or GFP-NuSAP after cold treatment. Cells were stained with an anti-α-tubulin antibody and DNA with Hoechst 33342. Scale bar, 5 μm. (**g**) Representative images of PAGFP α-tubulin stability in metaphase HeLa cells transfected with control siRNA, NuSAP siRNA, mCherry vector or mCherry-NuSAP after photoactivation. Dotted squares represent photoactivated region. Scale bar, 5 μm. (**h**) Bar chart of the half-lives of spindle MTs calculated with a double exponential regression fitting of photoactivation assays. *n* (control siRNA)=14 cells, *n* (NuSAP siRNA)=13, *n* (mCherry)=13, *n* (mCherry-NuSAP)=12. Error bars represent ±s.d. (**i**) FRAP assays; representative images of MT dynamics in metaphase stable mCherry-α-tubulin HeLa cells transfected with control siRNA, NuSAP siRNA, GFP-vector or GFP-NuSAP after photobleaching. Dotted squares represent the photobleaching region. Scale bar, 5 μm. (**j**) Dot plot representing the half-life of kinetochore microtubules (slow phase) and interpolar microtubules (fast phase) calculated according to a double exponential regression curve with FRAP assays in control siRNA-, NuSAP siRNA-, GFP-vector- or GFP-NuSAP-transfected metaphase cells. Mean±s.e.m. are indicated.

To validate our statistical analysis biologically, we conducted a microtubule gliding assay using purified Kid and NuSAP proteins (Supplementary Fig. 6a). As the kymographs shown in Fig. 7a,b, microtubules glided on the Kid-coated surface slowly. Kid was found to be a slow motile motor with a speed of 27.8±2.6 nm s^−1^, compared with the speed of K560 (311±23 nm s^−1^, Supplementary Fig. 6b). However, in the presence of NuSAP protein, the gliding speed was significantly enhanced (46.5±9.1 nm s^−1^; Fig. 7c and Supplementary Movie 6). These results indicate an important role of NuSAP on facilitating Kid motility *in vitro*, which further supports its role on Kid-generated PEF during chromosome oscillation in metaphase cells. To further confirm the regulation of NuSAP on Kid-generated PEF in cells, a double depletion of both NuSAP and Kid was conducted in both bipolar and monopolar cells (Supplementary Fig. 6c,d). Our results show that double depletion of NuSAP and Kid resulted in a severe decrease in oscillation amplitude and the velocity of the centre in both metaphase and monopolar cells (Fig. 7d,e). The chromosome behaviour in the double depletion of NuSAP and Kid cells is similar to that in the depletion of NuSAP or Kid cells (Figs 3, 4, 5, 6c,e), which proves that the function of NuSAP on the PEF conducted by Kid is critical for its role in chromosome oscillation in cells.

Our study also indicates a possible role of NuSAP on kMTs. Previous studies have indicated that NuSAP can stabilize microtubules and link them to chromosomes^14^,^15^,^16^. Thus, NuSAP may facilitate the PF generated by kMTs through its regulation of microtubule dynamics. To separate the role of NuSAP on kMTs, the cells were cold-treated. Cold-resistant kMTs in NuSAP-depleted cells were dramatically decreased, whereas a greater abundance of kMTs was observed in GFP-NuSAP-overexpressing cells (Fig. 7f). To better address this question, an fluorescence dissipation after photoactivation assay was performed (Fig. 7g). The KT region around the metaphase plate was photoactivated in PAGFP α-tubulin-expressing cells transfected with control siRNA, NuSAP siRNA, mCherry or mCherry-NuSAP, and the half-lives of kMTs were analysed with a double exponential equation^42^,^43^ (Fig. 7h). The increased half-lives of kMTs in NuSAP-overexpressed cells indicate that NuSAP functions as a kMT stabilizer in mitotic spindles. The effects of NuSAP on kMTs and iMTs were further deciphered using FRAP (fluorescence recovery after photobleaching) assays (Fig. 7i,j). The microtubule signal in the KT region was photobleached and the fluorescence intensity decay within the region was analysed. As shown in Fig. 7j, NuSAP regulates the stability of both kMTs and iMTs; however, the half-lives of the kMTs were more dependent on NuSAP compared with the half-lives of the iMTs. Our results indicate a function of NuSAP on kMT stability, which may explain its role on kMT-generated PF during chromosome oscillation in metaphase.

## Discussion

Here we show that the MAP, NuSAP, plays an important role in chromosome congression at metaphase. Previous studies have indicated that NuSAP is a microtubule stabilizer during mitosis, both *in vitro* and *in vivo*^14^,^15^,^16^. Our results extend these earlier findings by showing that NuSAP associates with the central spindle microtubules to regulate the alignment and orientation of the chromosome at the metaphase plate, facilitating proper chromosome congression during metaphase.

We further identify NuSAP as a novel regulator of the chromokinesin Kid. NuSAP associates with Kid to facilitate its distribution on microtubules. NuSAP and Kid are both expressed at high levels during mitosis^19^,^44^, and are regulated by the RanGTP gradient to accumulate at the metaphase plate^15^,^45^. We demonstrate that NuSAP binds Kid and plays a pivotal role in regulating Kid-specific functions during metaphase, and that this interaction facilitates the association of Kid with microtubules. The temporal and spatial nature of the regulation indicates that NuSAP actively regulates Kid during metaphase.

Kid functions as a key force-producing agent of the PEF^30^,^46^, and our quantitative live-cell imaging experiments show that NuSAP specifically regulates chromosome oscillation by governing the PEF generated by Kid. The effects of NuSAP depletion on the regulation of the amplitude and velocity of chromosome oscillation in both bipolar and monopolar cells correlate with those resulting from Kid depletion. The synergistic regulation of centromere movement by NuSAP with Kid occurs specifically at iMTs, as shown in the absence of kMTs following Nuf2 depletion. Our results suggest that the PEF produced by Kid depends significantly on the specific regulation by NuSAP. This is further supported by microtubule gliding assays using purified Kid and NuSAP proteins, which prove that full-length Kid is a motile motor with a slow speed (27.8 nm s^−1^). NuSAP positively facilitates microtubule gliding on Kid-coated surfaces, which further supports our analysis that NuSAP is important in the regulation of the Kid-generated PEF. Furthermore, to confirm our analysis in cell-based experiments, the double depletion of NuSAP and Kid was conducted and the results further evidence the major role of NuSAP on the Kid-generated PEF both in cells and *in vitro.* The differences between the double depletion of NuSAP/Kid from the depletion of NuSAP or Kid suggest other possible important functions of NuSAP on chromosome oscillation besides its regulation on Kid. Our results also indicate that NuSAP regulates the stability of iMTs, which might further influence the force generated by the dynamics of iMTs^37^. Other kinesins, for example, Kif4a (refs 7, 8) and dyneins^47^, involved in chromosome oscillation may be also regulated by NuSAP, and it would be interesting to study how other forces are influenced by NuSAP.

Our analysis also indicates that NuSAP has a moderate effect on the regulation of kMTs. We further examined the role of NuSAP in spindle microtubule stability and confirmed the function of NuSAP on both interpolar and kMT stability. The stability of kMTs is important for their ability to generate the PF. Thus, NuSAP may regulate the kMT-generated PF through its regulation on kMT stability. Our finding suggests that NuSAP may also influence the kMT-generated PF through the regulation of other motors on kMTs. Several other microtubule motors that play important roles in kMT dynamics and KT attachment may be involved in the PF generated by kMTs, for example, MCAK, which functions as a microtubule depolymerizer to regulate chromosome alignment and KT attachment^48^,^49^,^50^,^51^, and Kif18a, a microtubule plus-end-directed motor with depolymerase activity that has been implicated in proper chromosome oscillation^6^,^52^.

Using a simplified chromosome centre-projected approach, our data suggest that the average ICD and half-period did not change significantly in NuSAP- or Kid-depleted cells. However, the distribution of the ICD and half-period were influenced, and coupling of the ICD and velocity was abolished by the depletion of NuSAP or Kid. However, the complicated nature of chromosome oscillation means that it is possible that other factors might also be involved. For example, the stiffness of KTs absolves one portion of the unbalanced force on the chromosome during oscillation^9^,^11^,^36^,^53^, and changes in microtubule density in the absence of kMTs or in monopolar MT asters may also lead to changes in the PEF^2^,^4^,^9^,^37^. Further studies are needed to answer these questions.

In summary, our results show that NuSAP facilitates chromosome congression and interacts with Kid to regulate its localization during metaphase. Thus, NuSAP regulates chromosome oscillation through its regulations on both the stability of the kMTs and the Kid-generated PEF during metaphase.

## Methods

### Plasmids and siRNAs

The cDNAs of NuSAP, Kid and CENPA were amplified from a cDNA library extracted from HEK 293T cells and inserted into the pXJ40 vector tagged with GFP, mCherry, haemagglutinin (HA) or FLAG. His-NuSAP was inserted into the pET28b vector. GST-Kid was inserted into the pGEX-4T-1 vector. To construct Kid-GFP-His, Kid was first inserted into the EGFP-N1 vector and the Kid-GFP was then inserted into a modified pET32a vector with no other tags, which directly links Kid-GFP with the His tag followed by a termination codon. GFP-centrin1 was a gift from Dr Maki Murata-Hori. The mCherry α-tubulin was obtained from Addgene (#49149 (ref. 54)) and inserted into the pXJ40 vector tagged with GFP or HA. The K560-Dendra2 vector was a gift from Dr Jared C. Cochran. The sequences of NuSAP siRNA (5′- AAGCACCAAGAAGCTGAGAAT -3′ (ref. 14)), NuSAP siRNA No.2 (ref. 21; Silencer Selected 27674, Invitrogen-Life Technologies) and Nuf2 siRNA (5′- AAGCATGCCGTGAAACGTATA -3′ (refs 36, 39, 41, 55)) were described previously and synthesized by Sigma. The siRNA oligonucleotide for Kid was synthesized by Santa Cruz Biotechnology (#sc-44350) and Silencer Negative Control #1 siRNA was used as a control (Ambion).

### Cell culture and synchronization

HeLa and HEK 293T cells (American Type Culture Collection) were cultured in DMEM, supplemented with 10% fetal bovine serum (Gibco) and 1% penicillin/streptomycin at 37 °C, 5% CO_2_. Stable H2B-mCherry HeLa cells were generated using the pcDNA3-H2B-mCherry vector from Addgene (#20972 (ref. 56)) and cultured in the presence of 0.2 mg ml^−1^ G418 (Sigma). For DNA transfections, HEK 293T and HeLa cells were transfected using calcium phosphate and Effectene (Qiagen), respectively, to introduce target genes. For siRNA transfections, stable H2B-mCherry HeLa cells were transfected at 30–50% confluence, with 20 nM of each siRNA using Lipofectamine 2000 (Invitrogen) according to the manufacturer's protocol. For experiments involving double depletions, control cells were treated with 40 nM negative control siRNA. To synchronize cells at the G2/M stage, HeLa cells were treated with 100 ng ml^−1^ nocodazole (Sigma) for 16 h, washed three times with 1 × PBS and released into DMEM medium containing 10 μM MG132 (Sigma) for 2 h before fixation or live-imaging. For monopolar spindle experiments, cells were treated with 100 μM monastrol (Sigma) for 2 h before fixation or live-imaging. To analyse the interaction between NuSAP and Kid during the cell cycle, HeLa cells were treated with 2 mM thymidine (Sigma) for 24 h, released for 3 h and treated with 100 ng ml^−1^ nocodazole for 11 h, followed by 10 μM MG132 treatment for 2 h. Cells were released by washing three to five times with pre-warmed medium and plated into fresh DMEM, supplemented with 10% fetal bovine serum. Samples were taken at the indicated time points and processed for western blot analysis.

### Immunoprecipitation and western blot assays

For immunoprecipitation assays, HEK 293T and HeLa cells were lysed in M-PER (Thermo Scientific) with protease inhibitors, including 1 mM Na_3_VO_4_, 10 μg ml^−1^ aprotinin, 1 mM pepstatin, 1 mM leupeptin and 1 mM phenylmethylsulphonyl fluoride (PMSF). Nocodazole (10 μg ml^−1^) was added to completely depolymerize microtubules. FLAG M2 beads (20 μl; Sigma) were incubated with cell lysate for 1 h at 4 °C and washed three to five times in mammalian cell lysis buffer (50 mM HEPES, 100 mM NaCl, 1.5 mM MgCl_2_, 1 mM EDTA, 1% Triton X-100 and 10% glycerol). For endogenous immunoprecipitation assays, synchronized HeLa cells were shaken off and lysed. The cell lysate was incubated with 2 μg rabbit IgG (Santa Cruz Biotechnology), NuSAP antibody 1:1,000 (ab93779, Abcam) or Kid antibody 1:1,000 (ab75783, Abcam) bound to protein A-Sepharose beads (Amersham Biosciences) for 1 h at 4 °C. The immune complexes were washed three to five times in mammalian cell lysis buffer and applied to SDS-PAGE. Protein samples were separated on a 10% SDS gel and detected by the following primary antibodies: rabbit anti-FLAG 1:20,000 (F3165, Santa Cruz Biotechnology), rabbit anti-HA 1:2,000 (H6908, Santa Cruz Biotechnology), mouse anti-GAPDH 1:2,000 (sc32233, Santa Cruz Biotechnology), rabbit anti-cyclinB1 1:1,000 (H433, Santa Cruz Biotechnology), rabbit anti-Kid 1:1,000 (ab75783, Abcam), rabbit anti-NuSAP 1:1,000 (ab93779, Abcam) and mouse anti-NuSAP, which were detected by horseradish peroxidase-conjugated secondary antibodies (Santa Cruz Biotechnology).

### Protein expression and GST pull-down

GST-Kid or His-NuSAP were expressed in *Escherichia coli* BL21 (DE3) pLysS at 20 °C overnight after isopropyl-β-D-thiogalactoside (IPTG) induction. GST-Kid protein was purified with the use of glutathione-Sepharose 4B (Amersham Biosciences) and washed with wash buffer (50 mM Tris-HCl, 200 mM NaCl, 1 mM EDTA and 1 mM dithiothreitol (DTT)). His-NuSAP was resuspended in lysis buffer (10 mM imidazole, 50 mM NaH_2_PO_4_, 300 mM NaCl, 1 mM PMSF and protease inhibitor cocktail) and incubated with Ni-NTA agarose (Qiagen) at 4 °C for 1 h. His-NuSAP protein was eluted with elution buffer (250 mM imidazole, 50 mM NaH_2_PO_4_ and 300 mM NaCl) and stored at −80 °C. Kid-GFP-His and K560-Dendra2 were expressed in *E. coli* BL21 (DE3) pLysS at 16 °C overnight after IPTG induction. The bacterial cells were pelleted and resuspended in lysis buffer (20 mM imidazole, 100 mM NaCl, 1 mM MgCl_2_, 0.1 mM ATP, 1 mM PMSF and protease inhibitor cocktail). After sonication, the lysates were cleared by centrifugation and the supernatants were incubated with Ni-NTA resin for 1 h at 4 °C. Proteins were eluted with elution buffer containing 250 mM imidazole and dialysed into storage buffer (10 mM PIPES, 100 mM NaCl, 1 mM MgCl_2_, 0.01 mM ATP, 1 mM EGTA and 1 mM DTT). The protein was aliquoted and stored at −80 °C. The *in vitro* GST pull-down assay using purified proteins was performed in 50 mM Tris-HCl, pH 7.0, 150 mM NaCl, 1 mM EGTA and 0.5% Triton X-100, with 2 μg GST fusion protein and 2 μg target protein. After 3-h incubation at 4 °C, the GST beads were washed three times, and protein samples were separated using SDS-PAGE and analysed with Coomassie blue staining.

### Microtubule gliding assay

Motility assays were performed as previously described^57^. Rhodamine-labelled microtubules (Cytoskeleton) were polymerized in BRB80 buffer (80 mM PIPES, 1 mM MgCl_2_ and 1 mM EGTA) containing 1 mM GTP at 37 °C for 10 min. Flow chambers with a volume of ∼10 μl were constructed with adhesive tapes on glass slides and incubated with 1 mg ml^−1^ casein (Sigma) and kinesin (50 μM for Kid and 20 μM for K560). MTs were diluted in BRB80 buffer with 0.5 mg ml^−1^ casein, 22.5 mM glucose, 0.22 mg ml^−1^ glucose oxidase, 0.036 mg ml^−1^ catalase, 3 mM ATP and 10 μM taxol (Sigma), and were introduced into the flow chamber. MTs were incubated in the presence or absence of 20 nM NuSAP protein at room temperature for 5 min before flowing into the chamber. Unbound microtubules were washed off and images were collected near the middle of the flow cell using the Ultraview Vox Spinning disc confocal system (PerkinElmer).

### Immunofluorescence

HeLa cells were cultured on ethanol-sterilized coverslips in 12-well plates and were synchronized or monastrol-treated before fixation with ice-cold methanol at −20 °C for 10 min, followed by rehydration with 1 × PBS at room temperature for 10 min. The fixed cells were permeabilized with 0.3% Triton X-100 in 1 × PBS for 15 min and blocked with 2% bovine serum albumin for 45 min at room temperature. Mouse anti-α-tubulin 1:2,000 (T9026, Sigma), rabbit anti-γ-tubulin 1:200 (T5192, Sigma), human anticentromere antibody 1:2,000 (HSM0101, ImmunoVision), rabbit anti-Kid 1:200 (ab75783, Abcam) and rabbit anti-NuSAP 1:200 (ab93779, Abcam) were utilized to stain the respective proteins in cells. Alexa Fluor dye-conjugated goat anti-mouse, goat anti-rabbit or goat anti-human IgG (Invitrogen) were used as the secondary antibodies. DNA was stained with Hoechst 33342 (Invitrogen). Cells were mounted in FluorSave reagent (Calbiochem). The images were processed using either the Volocity software (PerkinElmer) or Image J (National Institutes of Health, Bethesda, MD), as required.

### Live-cell imaging

Stable H2B-mCherry HeLa cells were cultured on 35-mm glass-bottom Petri dishes (Greiner Bio-One) and synchronized or monastrol-treated before imaging. Images were collected at 37 °C using the Ultraview Vox Spinning disc confocal system (PerkinElmer), which includes a CSD-X1 spinning disk head (Yokogawa), a solid-state diode laser with both diode and diode pumped solid-state (DPPS) modules (25–50 mM) and an EMCCD camera C9100-50 (Hamamatsu). The images were taken with an Olympus Uplan SApo 100 × 1.4 oil lens controlled with the Volocity software (PerkinElmer).

Previous studies have indicated that the movement of centromeres at the periphery of the spindle is different from that of the KT in the middle^7^,^58^,^59^. The imaging conditions were based on the phototoxic effects on the cells and KT detection quality, in particular for the 3D live-cell imaging^36^,^60^,^61^; thus, only the centromeres in the middle region of the spindle were imaged and analysed. It has previously been suggested that to avoid aliasing in the analysis of motion characteristics, the motion should be sampled at least two times faster than its frequency^62^. The KT oscillation in HeLa cells is ∼60 s per period, which would require a sampling interval less than 30 s. To accommodate these requirements, 0.5 μm of each *z* section for 3 μm within the centre of the spindle was acquired every 15 s for 10 min. Only cells with spindles oriented parallel to the plane of focus were analysed and the focal plane at the centre of the spindle was chosen.

For FRAP experiments, stable mCherry-α-tubulin HeLa cells were synchronized at the metaphase stage and imaged in a 37 °C humid chamber supplied with 5% CO_2_ with a UltraVIEW Vox spinning disc confocal system (PerkinElmer). A 1 × 4 μm photobleaching spot was placed near the middle of the spindle. Fluorescence intensities were photobleaching- and background-corrected using the Volocity 3D imaging software (PerkinElmer). Double exponential regression analysis in the Sigmaplot software (Jandel Scientific) was utilized to fit the data to the equation *F*(*t*)=*A*_1_*e*^−*k*1*t*^+*A*_2_*e*^−*k*2*t*^, where *F*(*t*) stands for fluorescence intensity over time, and *k*_1_ and *k*_2_ represent the proportion of iMTs with a fast phase and kMTs with a slow phase, respectively. MT turnover half-life time was calculated by the equation *T*_1/2_=Ln2/*k* (refs 43, 63). For photoactivation, HeLa cells transfected with PAGFP-α-tubulin were synchronized and a 405-nm laser was focused on the selected 1 × 4 μm area for 1 s. Fluorescence intensity of the activated region was photobleaching- and background-corrected using Volocity (PerkinElmer), and the half-lives of kMTs and iMTs were analysed by double exponential fitting as described^42^,^43^,^64^.

### Quantitative analyses of 3D chromosome movements

The 3D images were first background-corrected with the background image in the Volocity software (PerkinElmer), and noise removal was achieved by convolving the raw images with the 3D Gaussian filter in Imaris (Bitplane). Chromosomes were tracked using the Imaris (Bitplane) 3D surface detection function, followed by manual corrections. The velocity and acceleration of chromosomes were analysed with Imaris using a centre projection function, and the images were processed with the Volocity software (PerkinElmer) and Image J (National Institutes of Health).

### Quantitative analyses of 3D centromere oscillation

To correctly detect the centromeres, the images were first background-corrected with the background image in the Volocity software (PerkinElmer), and noise removal was achieved by convolving the raw images with the 3D Gaussian filter in Imaris (Bitplane). Centromeres in bipolar metaphase cells and monopolar cells were tracked using the Imaris (Bitplane) 3D spot detection function, followed by manual corrections. The centromere trajectories were constructed using the single-particle tracking algorithm within the centre of the spindle to avoid track interruptions resulting from temporary centromere disappearance by movement out of the imaging volume, or detection failure. Tracking was detected with a Brownian motion model because centromere oscillation is neither linear nor contains any merging and splitting events. On the basis of the approximate parameters of the centromere movement, we set the maximum gap-closing time window as 1 and the search radius upper limit as 1.5. Only the tracks with more than 20 time points detected were analysed. To correct cell movement during the process of imaging, KT positions (*x*, *y*, *z*, *t*) were registered to the relative 3D position compared with spindle pole (*x*_o_, *y*_o_, *z*_o_, *t*) over time as (*x*−*x*_o_, *y*−*y*_o_, *z*−*z*_o_, *t*).

The 3D position information of each centromere track was extracted from Imaris, and the data were analysed with custom scripts in Perl (Perl Inc.) and run from the Perl command line. Sister pairing was based on the average distance and the distance variation using both 3D spatial and temporal information of the centromere tracks^35^. The average of the sister track pair should be closer and the distance variation within each track should be smaller, compared with other non-sisters. Thus, we calculated the average distance *d*_ij_ 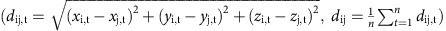 and the variance of the distance *v*_ij_ 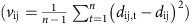 between each pair i *(x*_*i*_, *y*_i_, *z*_i_, *t*) and j (*x*_j_, *y*_j_, *z*_j_, *t*) tracks over the course of the imaging. The cost of the sister pairing considers both criteria together and was defined as *d*_ij_ × *v*_ij_. The acceptable bounds were set as 1.5, and the combination of the trajectory pairings was taken with the minimum global cost as the assignment of the sister tracks. Only paired tracks with over 20 time points matched out of the total 41 time points were analysed. The ICD was calculated as the average distance between the sister pairs 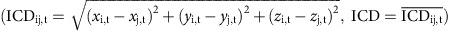.

The direction of the movement was set based on the change of the distances between each centromere in the sister tracks to the spindle pole (*x*_o_, *y*_o_, *z*_o_, *t*) during two time points 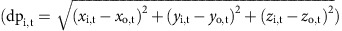. In bipolar cells, in a sister pair i (*x*_i_, *y*_i_, *z*_i_, *t*) and j (*x*_j_, *y*_j_, *z*_j_, *t*), for centromere i, which is closer to the specified spindle pole (dp_i_<dp_j_), the sign was set as positive (Sign_i,t_=+1) for movement to the pole (dp_i,t_>dp_i,t+1_) and negative (Sign_i,t_=−1) for movement away from the pole (dp_i,t_<dp_i,t+1_). For the other centromere, j, which is further from the specified spindle pole, the sign was the opposite (Sign_j,t_=+1) for movement away from the pole (dp_j,t_<dp_j,t+1_) and negative (Sign_j,t_=−1) for movement to the pole (dp_j,t_>dp_j,t+1_). In monopolar cells, the sign was set as positive (Sign_m,t_=+1) for movement to the pole (dp_m,t_>dp_m,t+1_) and negative (Sign_m,t_=−1) for movement away from the pole (dp_m,t_<dp_m,t+1_). The half-period was calculated as the time between two directional switching points based on the sign change (if Sign_switch+1_≠Sign_switch_, 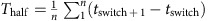).

The mimic centre track of sister pair i (*x*_i_, *y*_i_, *z*_i_, *t*) and j (*x*_j_, *y*_j_, *z*_j_, *t*) was set as ((*x*_i_+*x*_j_)/2, (*y*_i_+*y*_j_)/2, (*z*_i_+*z*_j_)/2, *t*). The oscillation amplitude was set as the distance of the projected centre moved between directional switch points 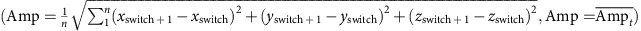. The average 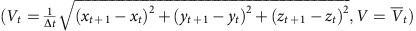 and the distribution of the centre velocity were calculated by the absolute value. The sign of the presented centre velocity over time shows the direction of the centre oscillation. The sign was set as positive (Sign_c,t_=+1) for movement to the pole (dp_c,t_>dp_c,t+1_) and negative (Sign_c,t_=−1) for movement away from the pole (dp_c,t_<dp_c,t+1_). The statistical analysis for calculation of the standard error of mean and confidence is described^62^ as 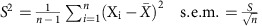.

## Additional information

**How to cite this article:** Li, C. *et al*. NuSAP governs chromosome oscillation by facilitating the Kid-generated polar ejection force. *Nat. Commun.* 7:10597 doi: 10.1038/ncomms10597 (2016).

## Supplementary Material

Supplementary Figures Supplementary Figures 1-6

Supplementary Movie 1 Representative 3D movie of chromosome movement in GFP-NuSAP-transfected metaphase cells. Stable mCherry-H2B HeLa cells were transfected with GFP-NuSAP and synchronised with nocodazole/MG132 before imaging. Frames were acquired at 15 s intervals for 10 min. The 3D view of both channels, the 3D view with the single chromosome marked, the GFP channel only and the 3D single chromosome movement tracks colour-coded for time are presented. This movie corresponds to Figure 1.

Supplementary Movie 2 Representative 3D movie of centromere oscillation in control siRNA, NuSAP siRNA and Kid siRNA depleted synchronised metaphase cells. Stable mCherry-H2B HeLa cells were transfected with GFP-CENPA to mark the centromeres and treated with control siRNA, NuSAP siRNA or Kid siRNA. The cells were synchronised with nocodazole/MG132 before imaging. Frames were acquired at 15 s intervals for 10 min. The 3D view, the 3D centromere tracks colour-coded for time, speed and acceleration, and a single 3D sister pair track colour-coded for speed and acceleration are presented. This movie corresponds to Figure 3.

Supplementary Movie 3 Representative 3D movie of centromere oscillation in Nuf2 siRNA, NuSAP/Nuf2 siRNA and Kid/Nuf2 siRNA depleted synchronised metaphase cells. Stable mCherry-H2B HeLa cells were transfected with GFP-CENPA to mark the centromeres and depleted with Nuf2 siRNA, NuSAP/Nuf2 siRNA and Kid/Nuf2 siRNA. The cells were synchronised with nocodazole/MG132 before imaging. Frames were acquired at 15 s intervals for 10 min. The 3D view, the 3D centromere tracks colour-coded for time, speed and acceleration, and a single 3D sister pair track colour-coded for speed and acceleration are presented. This movie corresponds to Figure 4.

Supplementary Movie 4 Representative 3D movie of centromere oscillation in control siRNA, NuSAP siRNA and Kid siRNA depleted monopolar cells. Stable mCherry-H2B HeLa cells were transfected with GFP-CENPA to mark the centromeres and depleted with control siRNA, NuSAP siRNA and Kid siRNA. The cells were treated with monastrol before imaging. Frames were acquired at 15 s intervals for 10 min. The 3D view, the 3D centromere tracks colour-coded for time, speed and acceleration, and a single 3D sister pair track colour-coded for speed and acceleration are presented. This movie corresponds to Figure 5.

Supplementary Movie 5 Representative 3D movie of centromere oscillation in Nuf2 siRNA, NuSAP/Nuf2 siRNA and Kid/Nuf2 siRNA depleted monopolar cells. Stable mCherry-H2B HeLa cells were transfected with GFP-CENPA to mark the centromeres and depleted with Nuf2 siRNA, NuSAP/Nuf2 siRNA and Kid/Nuf2 siRNA. The cells were treated with monastrol before imaging. Frames were acquired at 15 s intervals for 10 min. The 3D view, the 3D centromere tracks colour-coded for time, speed and acceleration, and a single 3D sister pair track colour-coded for speed and acceleration are presented. This movie corresponds to Figure 6.

Supplementary Movie 6 Representative movies of MT gliding on Kid-coated surface with or without NuSAP protein. Frames were acquired at 2 s intervals for 5 min. This movie corresponds to Figure 7.

## Figures and Tables

**Table 1:** Summary of the parameters defining centromere oscillation in bipolar and monopolar control, NuSAP, Kid, Nuf2, Nuf2/NuSAP and Nuf2/Kid siRNA cells.

**siRNA**	**Centre velocity (μm min**^**−1**^**)**	**Amplitude (μm)**	**Half-period (s)**	**ICD (μm)**	**No. of KT pairs/no. of cells**
*Bipolar*					
Control1*	*v*_1_=1.68±0.06	1.12±0.08	32.50±3.34	0.75±0.09	298/12
NuSAP	*v*_2_=1.39±0.03	0.78±0.11	32.56±3.55	0.69±0.09	226/8
Kid	*v*_3_=1.54±0.06	0.79±0.05	31.73±4.14	0.71±0.10	344/11
Nuf2	*v*_4_=1.73±0.06	1.15±0.08	33.48±6.55	0.61±0.13	467/15
NuSAP/Nuf2	*v*_5_=1.46±0.03	0.92±0.06	33.24±2.93	0.60±0.08	257/11
Kid/Nuf2	*v*_6_=1.59±0.04	0.95±0.06	33.34±2.89	0.58±0.10	238/9
Control2†	*v*_13_=1.70±0.11	1.18±0.12	33.83±4.36	0.73±0.08	263/9
NuSAP/Kid	*v*_14_=1.32±0.08	0.63±0.15	32.69±5.46	0.63±0.07	408/10
					
*Monopolar*					
Control1	*v*_7_=1.39±0.10	0.69±0.04	31.06±2.88	0.57±0.11	358/15
NuSAP	*v*_8_=1.25±0.06	0.66±0.04	30.65±2.87	0.56±0.07	261/10
Kid	*v*_9_=0.76±0.02	0.49±0.03	28.66±2.22	0.52±0.08	447/13
Nuf2	*v*_10_=1.58±0.09	0.83±0.05	29.97±3.04	0.52±0.09	341/15
NuSAP/Nuf2	*v*_11_=1.03±0.03	0.56±0.05	26.96±2.17	0.47±0.08	237/11
Kid/Nuf2	*v*_12_=1.21±0.03	0.66±0.08	29.01±2.30	0.50±0.08	357/12
Control2	*v*_15_=1.40±0.11	0.62±0.09	32.60±4.81	0.56±0.07	410/11
NuSAP/Kid	*v*_16_=0.74±0.04	0.47±0.10	30.35±4.05	0.49±0.07	384/12

ICD, intercentromere distance; Kid, kinesin-like DNA-binding protein; KT, kinetochore; NuSAP, nucleolar and spindle-associated protein; siRNA, short interfering RNA.

Parameter values are reported as the mean±s.e.m.

^*^Control1–scramble siRNA.

^†^Control2–double dose of scramble siRNA.
