# Supplementary material for: NuSAP governs chromosome oscillation by facilitating the Kid-generated polar ejection force
Source: Nat Commun. 2016 Feb 3;7:10597. doi: 10.1038/ncomms10597 (PMC4742958; doi:10.1038/ncomms10597)
Supplement: Supplementary Figures — 1-6 [file ncomms10597-s1.pdf]

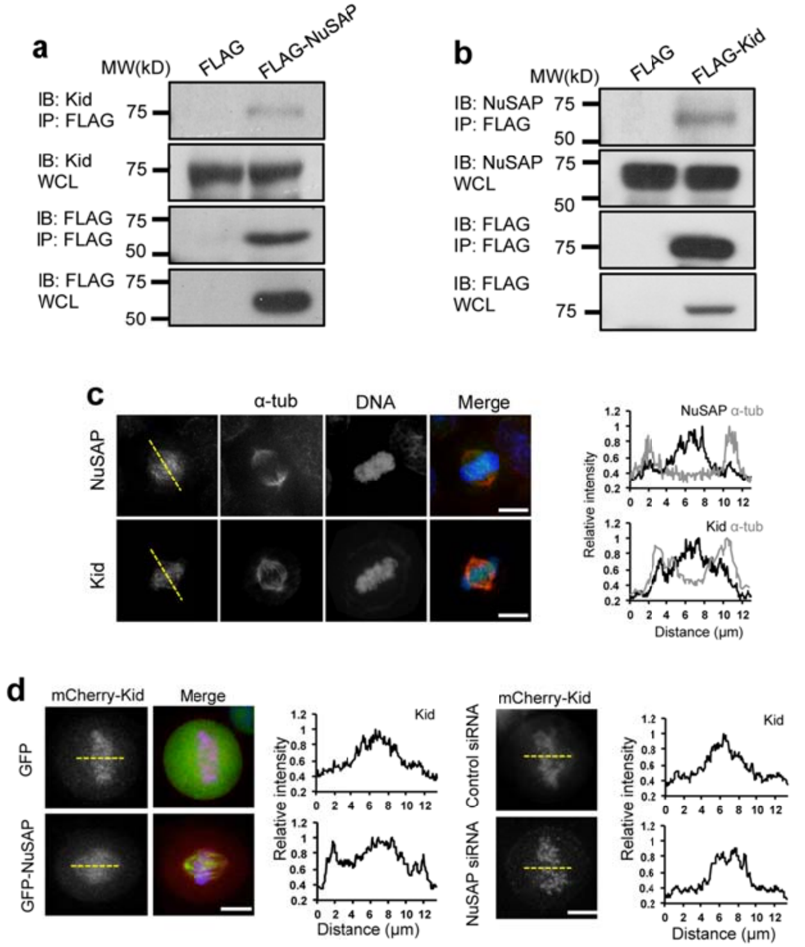

Supplementary Figure 1 NuSAP interacts with Kid and regulates its localisation during metaphase

(**a**) NuSAP immunoprecipitate contains Kid. FLAG and FLAG-NuSAP immunoprecipitates from 293T cell lysates were blotted for Kid. (**b**) The reverse immunoprecipitation of Kid contains NuSAP detected by NuSAP antibody. (**c**) Endogenous NuSAP or Kid localisation in metaphase HeLa cells with NuSAP or Kid antibody staining and the line profiles of NuSAP or Kid (black) along the metaphase spindle (grey). Mitotic spindles were labelled with anti-α-tubulin and the DNA with Hoechst 33342. Scale bar, 5 μm. (**d**) Kid localisation in live HeLa cells transfected with GFP-vector, GFP-NuSAP, control siRNA or NuSAP siRNA. Hoechst 33342 was added in the medium 5 min before imaging. The line profile of Kid localisation (black) is represented in the right graph. Scale bar, 5 μm.

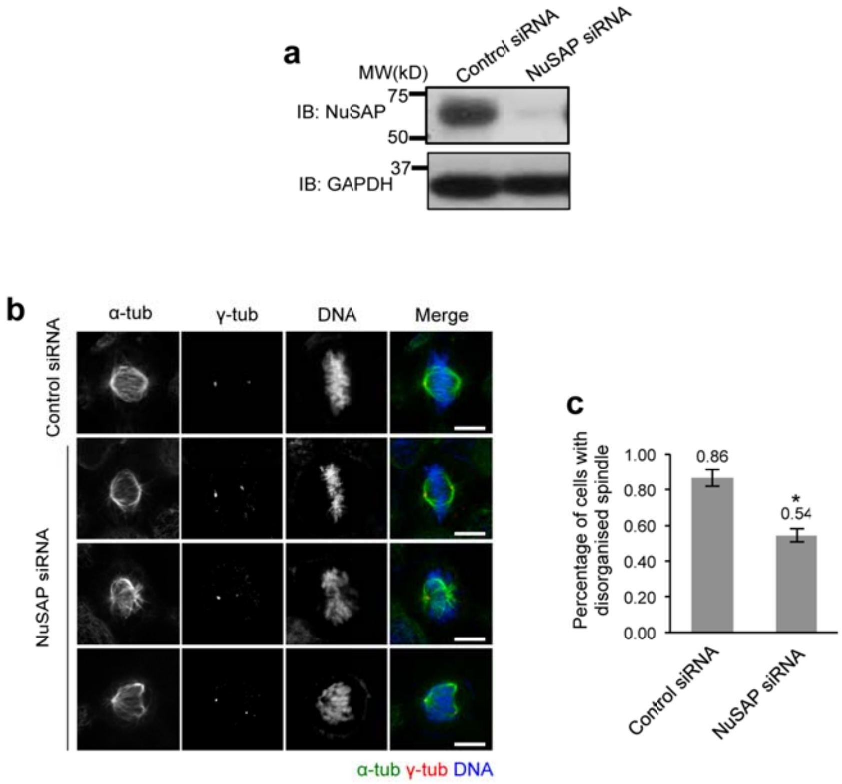

Supplementary Figure 2 Confirmation of NuSAP depletions by siRNA
(**a**) The effectiveness of NuSAP depletion in HeLa cells was analysed by western blot 48 h after siRNA treatment. The cell lysates were blotted for NuSAP and the amount of protein loading was detected using an anti-GAPDH antibody. (**b**) The metaphase spindles of control or NuSAP-depleted HeLa cells were visualised by immunofluorescence. HeLa cells were stained with anti-α-tubulin, anti-γ-tubulin and Hoechst 33342. Scale bar, 5 µm. (**c**) A bar chart represents the percentage of cells with disorganised spindle in control siRNA or NuSAP siRNA transfected metaphase HeLa cells. n(control siRNA)=37/3 independent experiments, n(NuSAP siRNA)=46/3. Error bars represents ±SD. * $p<0.001$.

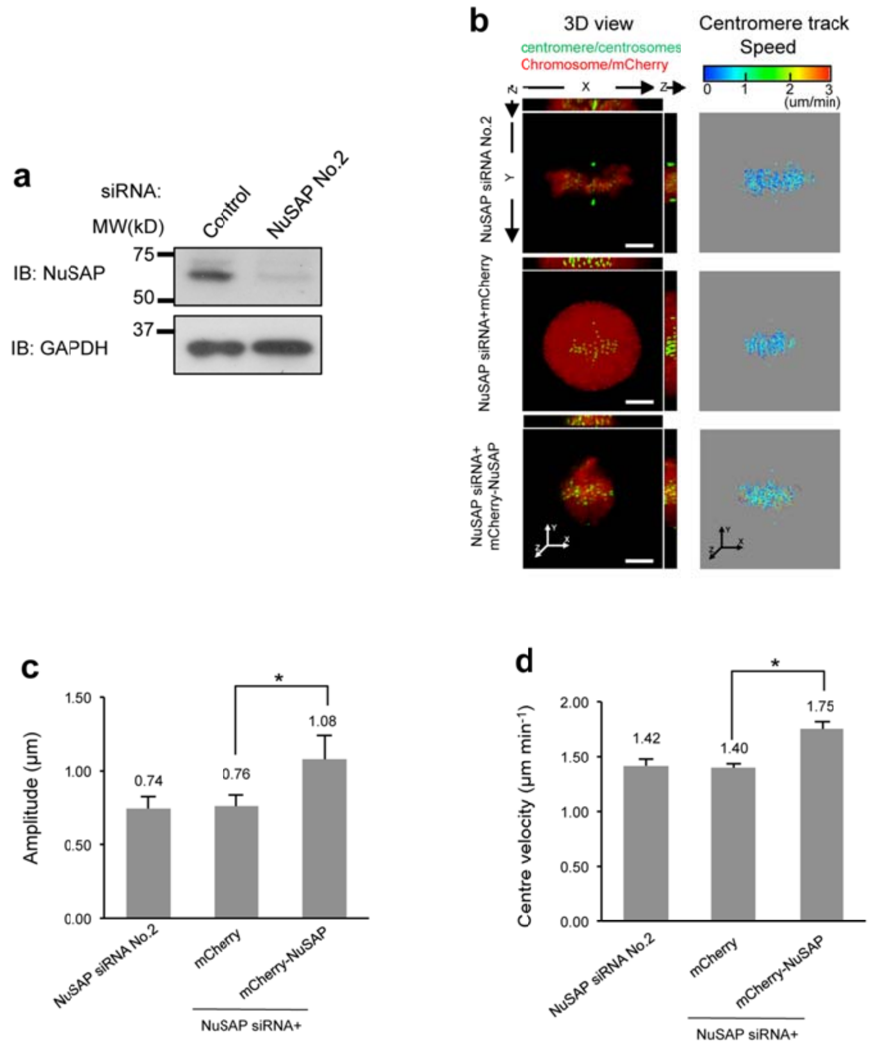

Supplementary Figure 3 NuSAP facilitates kinetochore oscillation during metaphase (**a**) The effectiveness of the second NuSAP siRNA was analysed by western blot 48 h after siRNA treatment. The cell lysates were blotted for NuSAP. The amount of protein loading was detected using an anti-GAPDH antibody. (**b**) Representative images of the 3D view and 3D centromere tracks colour-coded for velocity in NuSAP siRNA No.2, NuSAP siRNA with mCherry vector or mCherry-NuSAP transfected metaphase stable mCherry-H2B HeLa cells. The centromeres were marked with GFP-CENPA and centrosomes with GFP-centrin. Scale bar, 5 μm. (**c**-**d**) The bar charts represent the average of the amplitude (**c**) and centre velocity (**d**) of the centromere oscillation. n(NuSAP siRNA No.2)=215 KT pairs/10 cells, n(NuSAP siRNA+mCherry)=233/10, n(NuSAP siRNA+mCherry-NuSAP)=235/10. Error bars represent +SEM. * $p<0.001$.

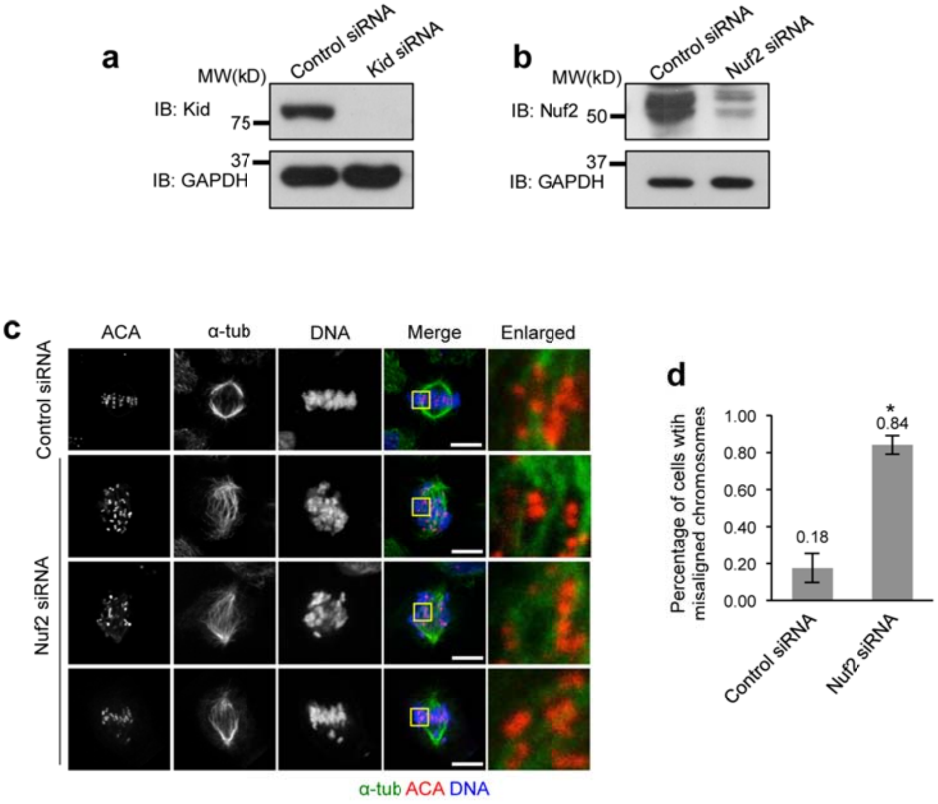

Supplementary Figure 4 Confirmation of Kid and Nuf2 depletions by siRNAs
(**a**-**b**) The effectiveness of Kid (**a**) and Nuf2 (**b**) depletion in HeLa cells were analysed by western blot 48 h after siRNA treatment. The cell lysates were blotted for Kid or Nuf2. The amount of protein loading was detected using an anti-GAPDH antibody. (**c**) The Nuf2 depletion in HeLa cells was visualised by immunofluorescence 48 h after siRNA treatment. HeLa cells were stained with ACA, anti-α-tubulin and Hoechst 33342. The detailed centromere localisation and kMT plus-ends attachment were enlarged in the right panel. Scale bar, 5 μm. (**d**) A bar chart represents the percentage of cells with misaligned chromosomes in control siRNA or Nuf2 siRNA transfected metaphase HeLa cells. n(control siRNA)=40/3 independent experiments, n(Nuf2 siRNA)=68/3. Error bars represents ±SD. * $p<0.001$.

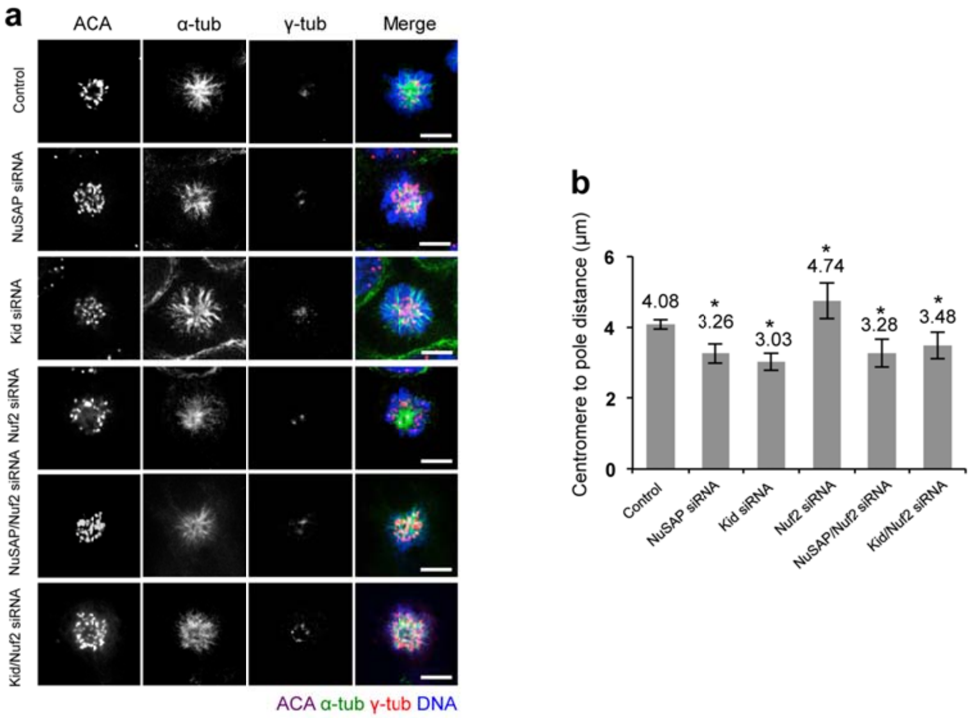

Supplementary Figure 5 NuSAP facilitates PEF generation together with Kid

(**a**) HeLa cells transfected with control siRNA, NuSAP siRNA or Kid siRNA with or without Nuf2 siRNA were treated with monastrol. Kinetochores were labeled with ACA, spindle poles with anti-γ-tubulin and DNA with Hoechst 33342. Scale bar, 5μm. (**b**) A bar chart represents the average distance from centromeres to spindle poles in monopolar HeLa cells. The number of centromere pairs quantified: n(Control siRNA)=57/3 independent experiments, n(NuSAP siRNA)=35/3, n(Kid siRNA)=37/3, n(Nuf2 siRNA)=37/3, n(NuSAP/Nuf2 siRNA)=42/3, n(Kid/Nuf2 siRNA)=45/3. Error bars represents ±SD. * $p<0.001$.

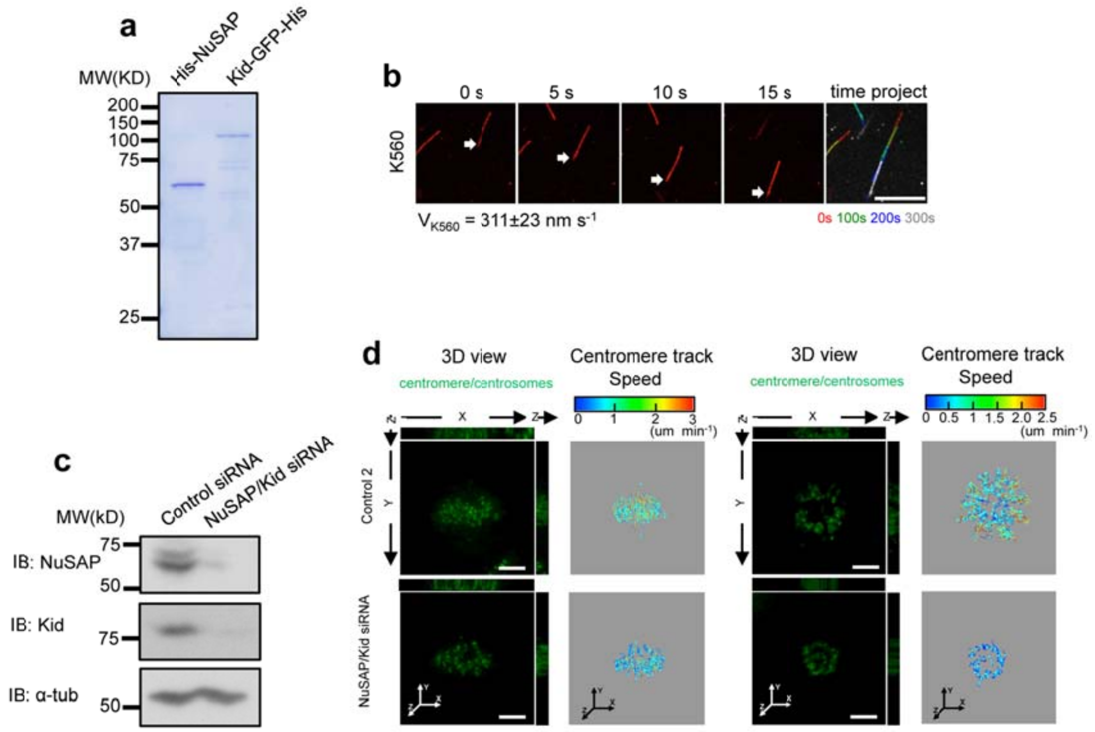

Supplementary Figure 6 NuSAP facilitates Kid gliding MTs *in vitro*

(**a**) Coomassie stained gels showing purifications of His-tagged NuSAP and Kid proteins from bacteria. (**b**) Representative images and colour project of MT gliding assay with K560 as control. Arrows represent one MT gliding at different time points. Images were acquired in a 2 s interval for 5 min. Scale bar, 3 μm. (**c**) The effectiveness of NuSAP and Kid depletion in HeLa cells were analysed by western blot 48 h after siRNA treatment. (**d**) Representative images of the 3D view and 3D centromere tracks colour-coded for velocity in control siRNA or NuSAP/Kid siRNA transfected metaphase cells. Scale bar, 5 μm.
